# Journalistic Enterprise

**Published:** 1889-03

**Authors:** 


### JOURNALISTIC ENTERPRISE.

The February and March numbers of the *Review* appear as one issue, and contains the full report of the anniversary meeting of the Chicago Dental Society. The volume contains 144 pages of reading matter, which was gotten out in the incredibly short space of two weeks' time, including, also, cuts illustrating the articles read. The work has been well, although hurriedly, done, and shows an amount of enterprise and push on the part of our western cotemporary that might well be emulated by some of our eastern journals. The present seems to be marking an era of advance in dental journalism that we herald as a harbinger of better things in the future. Some other of our cotemporaries have gotten a pointer or two the past year, and have made an effort to be more than recorders of society proceedings.

